# Tissue- and stage-specific Wnt target gene expression is controlled subsequent to β-catenin recruitment to cis-regulatory modules

**DOI:** 10.1242/dev.131664

**Published:** 2016-06-01

**Authors:** Yukio Nakamura, Eduardo de Paiva Alves, Gert Jan C. Veenstra, Stefan Hoppler

**Affiliations:** 1 Institute of Medical Sciences, Foresterhill Health Campus, University of Aberdeen, Aberdeen AB25 2ZD, UK; 2 Centre for Genome-Enabled Biology and Medicine, University of Aberdeen, Aberdeen AB24 3RY, UK; 3 Radboud University, Department of Molecular Developmental Biology, Faculty of Science, Radboud Institute for Molecular Life Sciences, 6500 HB Nijmegen, The Netherlands

**Keywords:** Wnt signalling, β-catenin, *Xenopus*, Gastrula, ChIP-seq, RNA-seq

## Abstract

Key signalling pathways, such as canonical Wnt/β-catenin signalling, operate repeatedly to regulate tissue- and stage-specific transcriptional responses during development. Although recruitment of nuclear β-catenin to target genomic loci serves as the hallmark of canonical Wnt signalling, mechanisms controlling stage- or tissue-specific transcriptional responses remain elusive. Here, a direct comparison of genome-wide occupancy of β-catenin with a stage-matched Wnt-regulated transcriptome reveals that only a subset of β-catenin-bound genomic loci are transcriptionally regulated by Wnt signalling. We demonstrate that Wnt signalling regulates β-catenin binding to Wnt target genes not only when they are transcriptionally regulated, but also in contexts in which their transcription remains unaffected. The transcriptional response to Wnt signalling depends on additional mechanisms, such as BMP or FGF signalling for the particular genes we investigated, which do not influence β-catenin recruitment. Our findings suggest a more general paradigm for Wnt-regulated transcriptional mechanisms, which is relevant for tissue-specific functions of Wnt/β-catenin signalling in embryonic development but also for stem cell-mediated homeostasis and cancer. Chromatin association of β-catenin, even to functional Wnt-response elements, can no longer be considered a proxy for identifying transcriptionally Wnt-regulated genes. Context-dependent mechanisms are crucial for transcriptional activation of Wnt/β-catenin target genes subsequent to β-catenin recruitment. Our conclusions therefore also imply that Wnt-regulated β-catenin binding in one context can mark Wnt-regulated transcriptional target genes for different contexts.

## Abstract (precis)

**Highlighted article:** Dual ChIP-seq and RNA-seq *in vivo* experiments show that the context-specific events that occur subsequent to β-catenin binding enable gene-specific regulation, rather than β-catenin recruitment *per se*.

## INTRODUCTION

Key signalling mechanisms are deployed repeatedly during embryonic development to regulate differential gene expression, often in combination with each other and with other regulatory mechanisms. Wnt/β-catenin signalling (hereafter referred to as Wnt signalling) is an important, evolutionarily conserved cell-to-cell signalling mechanism that regulates the transcription of specific target genes (reviewed by Cadigan and Waterman, 2012; Hoppler and Nakamura, 2014). Wnt signalling operates repeatedly during embryogenesis, in stem cell-mediated homeostasis and in cancer (reviewed by Hoppler and Moon, 2014; Nusse et al., 2012). The textbook view asserts that activation of the canonical Wnt signalling pathway causes β-catenin stabilisation and nuclear localisation, where β-catenin associates with TCF/LEF transcription factors bound to so-called Wnt-response DNA regulatory elements (WREs) to activate the transcription of nearby Wnt target genes (reviewed by Nusse, 2012). Recruitment of nuclear β-catenin to target chromatin regions is therefore thought to be the critical step for Wnt-regulated target gene regulation. However, the developmental, cellular and transcriptional responses to Wnt signalling are often remarkably specific for particular stages, tissues and cell lineages, and the molecular mechanisms by which the specific Wnt/β-catenin target genes are regulated in different cellular and developmental contexts are still largely unknown. Characterising these context-specific mechanisms is therefore important for understanding the specific functional roles of Wnt signalling in embryonic development and disease.

Early embryos represent ideal experimental models for studying the fundamental molecular mechanisms by which Wnt signalling regulates such context-specific responses, since there are rapid and fundamental changes in the cellular and developmental response to Wnt signalling (reviewed by Zylkiewicz et al., 2014). This is particularly prominent in the early *Xenopus* embryo (Fig. S1): maternally activated Wnt signalling before the general onset of zygotic transcription at the mid-blastula transition (MBT) (Newport and Kirschner, 1982) regulates specific genes that then function to establish dorsal development (e.g. Funayama et al., 1995; Heasman et al., 2000; McMahon and Moon, 1989); but, only shortly thereafter, early zygotic Wnt signalling promotes ventral development (Christian and Moon, 1993; Hoppler et al., 1996); yet, both are mediated by the β-catenin-dependent pathway (Hamilton et al., 2001). This radical change in the stage-specific response to Wnt signalling makes *Xenopus* embryos a unique model for dissecting the molecular mechanisms that determine context-specific responses to Wnt signalling. Direct target genes of maternally activated Wnt signalling have been described (e.g. Blythe et al., 2010; Brannon et al., 1997; Crease et al., 1998; Laurent et al., 1997); however, genes specifically regulated by early zygotic Wnt signalling are much less well understood. Identifying such direct Wnt target genes would not only be informative concerning the gene regulatory network that operates in the ventrolateral prospective mesoderm, but also more generally concerning the fundamental molecular mechanisms of context-specific Wnt target gene regulation.

Here, we report genome-wide identification of such stage-specific Wnt target genes through β-catenin chromatin immunoprecipitation followed by high-throughput sequencing (ChIP-seq) combined with RNA sequencing (RNA-seq) analysis of the relevant Wnt-regulated transcriptome. Although the early *Xenopus* embryo shows β-catenin occupancy at many genomic loci, our analysis reveals that transcriptional expression is Wnt regulated at only a subset of these loci. Thus, Wnt-regulated β-catenin recruitment to gene loci is required, but not sufficient, for Wnt target gene expression. We find instead that the tissue- and stage-specific context can regulate Wnt target gene expression subsequent to β-catenin recruitment to cis-regulatory modules at these loci.

## RESULTS

### Genome-wide mapping of β-catenin association in *Xenopus* early gastrulae

Nuclear localisation of β-catenin is the hallmark of canonical Wnt signalling (Schneider et al., 1996; Schohl and Fagotto, 2002). In the nucleus, β-catenin regulates target gene expression in association with DNA-binding proteins, particularly those of the TCF/LEF family (reviewed by Cadigan and Waterman, 2012; Hoppler and Waterman, 2014). β-catenin ChIP-seq analysis had been used to identify direct transcriptional targets of Wnt signalling in cancer tissue and cultured cells (Bottomly et al., 2010; Park et al., 2012; Schuijers et al., 2014; Watanabe et al., 2014). We therefore reasoned that β-catenin ChIP-seq analysis in intact gastrula stage *Xenopus tropicalis* embryos would identify early gastrula-specific Wnt target genes.

**Fig. 1.**
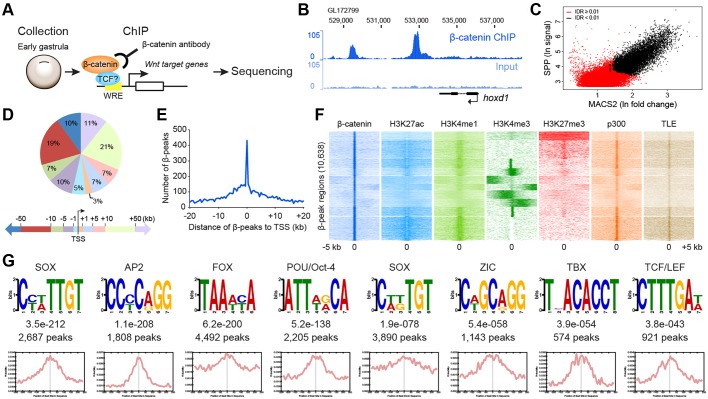
**β-catenin ChIP-seq analysis of *Xenopus* early gastrulae.** (A) Experimental design of β-catenin ChIP-seq analysis. Early gastrulae were collected and fixed. Following chromatin shearing, β-catenin antibodies were used to selectively precipitate DNA fragments bound by β-catenin-containing protein complexes. Subsequently, the precipitated DNA fragments were sequenced. (B) Genome view of example β-catenin target gene *hoxd1*. Note the clear β-catenin ChIP-seq peaks (β-peaks) downstream (to the left) of the *hoxd1* locus. (C) Scatter plot combining peak calling analysis by SPP [considering signal strength, applying false discovery rate (FDR)≤0.1] and MACS2 (considering fold change, applying *P*≤0.01) software, with black dots indicating 10,638 β-peaks reproducibly called [applying an irreproducible discovery rate (IDR)≤0.01]. (D,E) β-peaks are associated with sequences throughout the genome (D) but enriched close to and just upstream (putative promoter) of the transcriptional start site (TSS) of nearby genes (E; analysed in 500 bp bins). Pie chart (D) shows the percentage of β-peaks according to their location relative to TSS (within 1 kb, 1-5 kb, 5-10 kb, 10-50 kb, over 50 kb upstream or downstream of TSS). (F) Heat map illustrating genome-wide association of β-peaks with histone modifications and transcription co-factor binding sites indicative of cis-regulatory modules (CRMs; such as promoters and enhancers) in patterns that can be clustered into ten groups. Each horizontal line represents the 5 kb downstream and upstream regions of ChIP-seq data around a β-peak. (G) Enriched motifs from *de novo* motif search of sequences under β-peaks. Note the identification of consensus TCF/LEF binding but also other known transcription factor binding motifs. Statistical significance (e-values) and the number of β-peaks are indicated below each motif logo. The analysis of motif distribution shows central enrichment of motifs within β-peak regions (500 bp window).

We developed a reliable β-catenin ChIP protocol for analysis at the early gastrula stage (stage 10.25, Fig. 1A, Fig. S2) by optimising first chromatin shearing conditions for fragments of ∼200 bp (Fig. S2A), then the immunoprecipitation of chromatin-associated β-catenin protein with two different β-catenin antibodies, as well as IgG as a negative control (see Materials and Methods). Specific binding of β-catenin by the antibodies was validated by western blotting and also by β-catenin ChIP-qPCR (Fig. S2B-D). In the ChIP-qPCR validation, we analysed known WREs in genes known to be Wnt regulated at this stage [*hoxd1* (Janssens et al., 2010) and *msgn1* (Wang et al., 2007)] as positive controls, and genomic regions not containing WREs (from *odc1* and *hoxd1*) as negative controls. ChIP DNA samples and input control DNA samples were each pooled from three validated ChIP experiments and sequenced.

Clear β-catenin ChIP-seq peaks (hereafter referred to as β-peaks) were found at known direct Wnt target loci in the *X*. *tropicalis* genome [e.g. the *hoxd1* locus (Janssens et al., 2010), Fig. 1B]. The β-catenin ChIP-seq also confirmed no β-catenin association at the negative control *odc1* locus (data not shown). Two independent peak-calling algorithms followed by stringent irreproducible discovery rate (IDR) analysis (Li et al., 2011) identified 10,638 reproducible β-peaks across the *X*. *tropicalis* genome (Fig. 1C), which can be assigned to 5193 genes (Table S1). β-peaks are widely distributed throughout the genome, close to and further away from the transcriptional start site (TSS) of annotated genes (Fig. 1D), but we find an enrichment close to and just 5′ of the TSS of genes (Fig. 1E) and also a genome-wide correlation with putative cis-regulatory sequences, such as promoters, enhancers or silencers, which are collectively referred to here as cis-regulatory modules (CRMs) [Fig. 1F; data for H3K4me3 and H3K27me3 from Akkers et al. (2009), representing active promoters and inactive chromatin states, respectively; data for H3K27ac and H3K4me1 (both indicating active enhancers), for the transcriptional co-activator p300 and for the transcriptional co-repressor Transducin-like enhancer of split (TLE; also known as Groucho in *Drosophila*) from Yasuoka et al. (2014)]. For instance, correlation of β-catenin with p300-associated and with TLE-associated sites was 47.4% and 86.4%, respectively. We sought to detect enriched DNA sequences shared among the identified β-peaks by performing a *de novo* motif search on all β-peaks (Fig. 1G). As expected, consensus TCF/LEF core binding sequences were identified. Additionally, other known transcription factor binding motifs were found, some of which had also been identified in previous β-catenin ChIP-seq studies (Schuijers et al., 2014; Zhang et al., 2013) (see Discussion).

### RNA-seq analysis of the *wnt8a*-regulated transcriptome

**Fig. 2.**
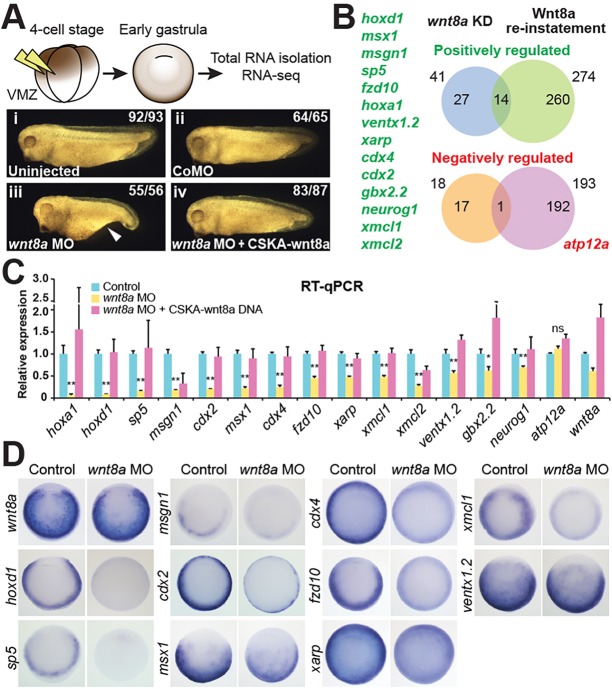
**RNA-seq analysis to identify *wnt8a*-regulated genes.** (A) Experimental design to identify *wnt8a*-regulated genes. *wnt8a* MO and a standard control MO (CoMO) were microinjected into the ventral marginal zone (VMZ) of four-cell stage embryos (prospective endogenous *wnt8a*-expressing and ventral mesoderm tissue). For the reinstatement experiment, *wnt8a* MO was co-injected together with a DNA construct driving exogenous Wnt8a (CSKA-wnt8a) in the same tissue. Three biological replicates per experimental sample were sequenced. The experimental conditions were optimised by comparing the morphology of (i) uninjected embryos with the embryos injected with (ii) CoMO, (iii) *wnt8a* MO or (iv) *wnt8a* MO plus CSKA-wnt8a DNA, as well as expected changes in expression of candidate genes (Fig. S3). (B) Venn diagrams of genes that are positively (top) or negatively (bottom) regulated by Wnt8a signalling identified by generalised linear model (GLM) statistical analysis (FDR<0.1; see the supplementary Materials and Methods) of RNA-seq results. Forty-one genes were identified with reduced expression in the *wnt8a* knockdown (KD; blue, compared with uninjected and CoMO-injected controls) and 274 genes with increased expression when Wnt8a expression was reinstated (green, compared with *wnt8a* knockdown). A shortlist of 14 *wnt8a* positively regulated genes (listed on the left) was selected for further analysis by the overlap between these two groups of genes (see Table S2 for full gene lists). Eighteen genes with increased expression were identified in the *wnt8a* knockdown (amber) and 193 genes with reduced expression when Wnt8a expression was reinstated (purple), with one gene (*atp12a*) in the overlap and therefore apparently negatively regulated by *wnt8a*. (C) Validation of RNA-seq-discovered candidate genes by RT-qPCR. Transcripts collected from embryos microinjected into all four blastomeres with *wnt8a* MO, or with *wnt8a* MO co-injected with CSKA-wnt8a DNA, were compared with control (CoMO injected). All 14 positively *wnt8a*-regulated candidate genes of the shortlist were confirmed; but not *atp12a*, which had been suggested to be negatively regulated. Note the varying extent of dependence on *wnt8a* function for the different genes. *P<0.1, **P<0.05; ns, not significant (*P*≥0.1); two-tailed Student's *t*-test. Error bars represent s.d. of two biological replicates. (D) Vegetal view of early gastrulae (with dorsal up) of control (uninjected) and *wnt8a* MO-injected embryos. Note the expression of *wnt8a*-regulated genes in a similar, but not always identical pattern, as *wnt8a.* Also note the reduced expression to varying extents in *wnt8a* MO-injected embryos.

Independently, we performed transcriptome analysis using RNA-seq in order to identify Wnt-regulated transcripts at the early gastrula stage. Early zygotic Wnt signalling is activated in prospective ventral mesoderm by *wnt8a*, which is the predominant Wnt gene expressed during later blastula stages (Christian et al., 1991; Collart et al., 2014). We developed an experimental design that allowed us to identify genes regulated by Wnt8a signalling (*wnt8a*-regulated genes, Fig. 2A). We compared the mRNA expression profile a few hours after the onset of zygotic transcription at early gastrula (stage 10.25) in two control conditions with that in two experimental conditions: embryos in which endogenous *wnt8a* was knocked down with a previously validated antisense morpholino oligonucleotide (MO) (Rana et al., 2006); and the same *wnt8a* knockdown embryos but with experimentally reinstated expression with an MO-insensitive, Wnt8a-expressing DNA construct (Fig. S3A).

We initially optimised the experimental conditions so that the *wnt8a* knockdown not only consistently caused the well-established *wnt8a* loss-of-function morphological phenotype (Hoppler et al., 1996; Rana et al., 2006), but also is then substantially rescued to normal embryonic morphology by our experimentally targeted reinstatement of stage-specific Wnt8a expression (Fig. 2A) (Christian and Moon, 1993). We confirmed that the morphological changes caused by the knockdown and reinstatement of Wnt8a expression are accompanied by predicted changes in the expression of previously reported *wnt8a*-regulated genes (Fig. S3B,C). In addition, unaltered gene expression levels of the well-known maternal Wnt target gene *siamois* (*sia1*) (Fig. S3C) confirmed that our experimental manipulation at cleavage stages (MO and DNA microinjection) does not affect early gene regulatory and dorsal axis establishment processes controlled by maternal Wnt signalling (see below).

Statistical analysis of the RNA-seq results, applying a generalised linear model (GLM) (Anders and Huber, 2010), identified an initial longlist of 274 genes potentially positively regulated and 193 genes potentially negatively regulated by *wnt8a* (Fig. 2B, seeTable S2). As expected, this list includes previously identified Wnt-regulated genes, such as *axin2*/*xarp* (Hufton et al., 2006), *hoxd1* (Janssens et al., 2010), *sp5* (Weidinger et al., 2005) and *ventx1* (Hoppler and Moon, 1998). However, also included are genes with relatively subtle changes in gene expression, which might not be physiologically relevant for embryonic development. In order to create a more manageable shortlist for further detailed analysis we decided to focus on genes that were significantly affected by both knockdown and reinstatement of Wnt8a expression (Fig. 2B). This resulted in a shortlist of 14 high-confidence *wnt8a* positively regulated genes, which have reduced expression in *wnt8a* knockdown and are increased upon Wnt8a reinstatement. This included two uncharacterised genes (*ENSXETG00000010483* and *ENSXETG00000030701*), which showed strong sequence similarity to each other and resembled *Xenopus laevis marginal coil* (*xmc*, Fig. S4). We therefore named *ENSXETG00000010483 xmc-like 1* (*xmcl1*) and *ENSXETG00000030701 xmc-like 2* (*xmcl2*). Applying the same restrictive criteria for shortlisting suggested only one gene, *apt12a*, to be negatively regulated by Wnt8a signalling (Fig. 2B).

All 14 *wnt8a* positively regulated genes were successfully validated (Fig. 2C). They were all shown to be expressed at the early gastrula stage when assayed by quantitative reverse transcription PCR (RT-qPCR) and, as expected, their expression was dependent on *wnt8a* function, although clearly to different degrees. However, the one gene that was apparently negatively regulated could not be validated. Therefore, consistent with the expected major role of Wnt signalling, we find that Wnt8a signalling mainly positively controls gene expression in early gastrula embryos and we proceeded to focus on *wnt8a* positively regulated genes. Expression of ten of the *wnt8a* positively regulated genes was detectable by whole-mount RNA *in situ* hybridisation in a pattern consistent with the expected signalling range of *wnt8a*-expressing cells mostly in the ventral and lateral prospective mesoderm, and, additionally, this expression was confirmed to be dependent on *wnt8a* function, but again clearly to varying degrees (Fig. 2D).

### Identification of direct *wnt8a* target gene loci

**Fig. 3.**
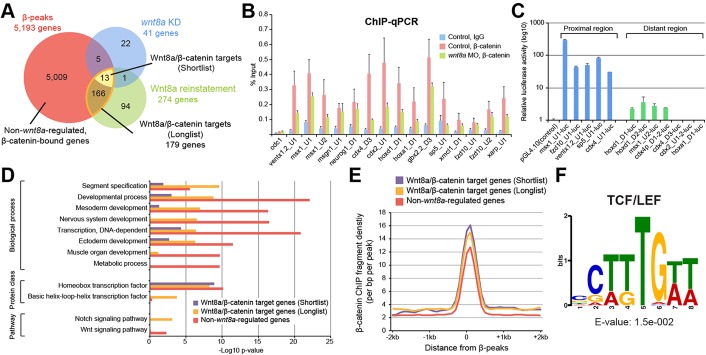
**Integrating β-catenin ChIP-seq and RNA-seq analysis to identify direct Wnt8a/β-catenin target genes.** (A) Venn diagram illustrating overlap between genes near β-peaks (red) and the *wnt8a* positively regulated genes (as in Fig. 2B). Note that from among the longlist of 274 potential *wnt8a*-regulated genes, 179 are associated with identified β-peaks (amber border around lens-shaped area), representing the longlist of probable direct Wnt8a/β-catenin target genes. Also note that all but one (*xmcl2*) of the validated shortlist of *wnt8a* positively regulated genes are among these and therefore represent the shortlist of 13 direct Wnt8a/β-catenin target genes (yellow). Also note that the majority of gene loci near β-peaks are not correlated with *wnt8a*-regulated genes and, conversely, that more than one-third of *wnt8a*-regulated genes in the longlist are not associated with identified β-peaks (most likely representing indirect *wnt8a* targets). (B) β-catenin ChIP-qPCR of identified β-peaks of our shortlist in chromatin extracted from control (uninjected) and *wnt8a* MO-injected embryos. Note that β-catenin association is reduced in the *wnt8a* loss-of-function experiment for most of the 15 β-peaks analysed. IgG antibodies were used as control. Error bars represent s.e.m. of three to five biological replicates. (C) Luciferase assays of reporter constructs containing sequences near identified β-peaks of *wnt8a*-regulated genes. Error bars represent s.d. of three biological replicates. (D) GO analysis suggests that β-peak-associated genes tend to encode transcription factors and also cell-to-cell signalling components, and to function in developmental processes, with different emphasis between *wnt8a*-regulated (purple and amber) and non-regulated genes (red). (E) DNA occupancy level of β**-**catenin around the peak summit shows higher enrichment in direct Wnt8a/β-catenin target gene loci [shortlist (purple) and longlist (amber)] compared with non-*wnt8a*-regulated genes (red). Read density was analysed using HOMER (bin size 100 bp). (F) TCF/LEF consensus motif is enriched under all 58 β-peaks associated with all 13 shortlisted Wnt8a/β-catenin target genes.

By combining the β-catenin ChIP-seq and the *wnt8a*-regulated transcriptome datasets, we identified 13 from our shortlist of 14 and 179 from our longlist of 274 *wnt8a*-regulated genes among the 5193 genes associated with β-peaks (Fig. 3A; see also examples in Fig. S5 and Table S3). By definition, we considered these 13 and 179 genes as our shortlist and longlist of direct Wnt8a/β-catenin target genes, respectively (Table S3).

We performed ChIP-qPCR analysis to examine whether Wnt8a signalling, as expected, controls β-catenin recruitment to the CRMs of Wnt8a/β-catenin targets. Knockdown of endogenous *wnt8a* resulted in reduction of β-catenin binding compared with the control, confirming that β-catenin association with these 13 shortlisted *wnt8a* target gene loci was dependent on *wnt8a* function (Fig. 3B). To assess the transcriptional activity of the β-peaks, we selected five β-peak elements from proximal regions just upstream of the TSS and seven from more distant regions, and tested them in luciferase reporter assays (Fig. 3C). All β-peak sequences from proximal regions strongly induced expression of the luciferase reporter (greater than 10-fold compared with a control vector), and four out of the seven distant β-peak sequences activated a heterologous basal promoter driving luciferase expression with weaker activity. Taken together, these results support the conclusion that the identified β-peak genomic regions control β-catenin-mediated transcription in response to Wnt8a signalling.

Approximately one-third of apparently *wnt8a*-regulated genes were devoid of any identifiable associated β-peak. These 94 genes were found in the Wnt8a reinstatement condition and might therefore be expressed due to Wnt8a overexpression. They might represent genes indirectly regulated by Wnt/β-catenin signalling or by β-catenin-independent Wnt signalling mechanisms, but we did not analyse them further in the current study. Because Wnt8a signalling in the prospective ventral mesoderm is mediated by the β-catenin-dependent pathway (Hamilton et al., 2001), we instead investigated two classes of β-catenin-associated genes: the direct Wnt8a/β-catenin target genes described above (i.e. the 13 and 179 genes of the shortlist and longlist, respectively) and 5009 β-catenin-bound but non-*wnt8a*-regulated genes (see also examples in Fig. S5E,F). We anticipated that comparing these two classes of genes would provide additional insight into how Wnt/β-catenin target genes are regulated.

First, we performed gene ontology (GO) analysis to identify whether these different classes are predicted to function in different biological processes (Fig. 3D). Our analysis showed, however, that the different classes are enriched for similar developmental processes, such as mesoderm development, and also that they both mainly contain genes encoding transcription factors, such as homeobox genes. Despite these similarities, they show some differences (compare purple and amber with red bars in Fig. 3D); in particular, the non-*wnt8a*-regulated genes show an even higher association with metabolic and later developmental processes (e.g. muscle, neural and non-neural ectoderm development) (see Discussion).

Next, in order to identify context-specific Wnt signalling mechanisms, we characterised the genomic sequences under the β-peaks of Wnt8a/β-catenin target genes when compared with β-catenin-bound but non-*wnt8a*-regulated genes. As shown above, all β-peaks were generally found to be enriched around TSSs (Fig. 1E); however, compared with non-*wnt8a*-regulated genes (10.1%), we found that *wnt8a*-regulated genes (30.7%) and particularly our shortlist (53.8%) exhibited an even higher enrichment of β-peaks within 1 kb regions upstream of the TSS. In addition, Wnt8a/β-catenin target genes tend to have more clearly defined β-peaks than the non-*wnt8a*-regulated gene class (Fig. 3E; see examples in Fig. S5A,B,E,F). Therefore, these two classes of β-catenin-associated genes exhibit subtly different levels and relative genomic locations of β-catenin recruitment. However, *de novo* motif discovery among β-peaks associated with these *wnt8a*-regulated or non-regulated genes uncovered essentially the same motifs among their β-peaks (Fig. S6). Therefore, these other motifs in *wnt8a*-regulated and non-regulated genes appear to exist more generally in β-peaks, implying they are not involved in regulating context-specific *wnt8a* target gene expression. Interestingly, the TCF/LEF motif was the only shared sequence motif found among all 13 genes of the shortlist (Fig. 3F), suggesting that TCF/LEF motif-dependent actions might constitute the only shared mechanism regulating context-specific *wnt8a* target gene expression (see below).

Together, this analysis suggests subtle quantitative, but no obvious qualitative, differences between *wnt8a*-regulated and non-*wnt8a*-regulated β-catenin-associated loci.

### β-catenin-chromatin association is not sufficient for transcriptional regulation of direct Wnt target genes

We had discovered β-catenin-associated loci in gastrula embryos that were not transcriptionally regulated by *wnt8a* function. We speculated whether these β-catenin-associated loci could represent Wnt target genes regulated in other tissues and at other stages. Conversely, we wondered whether our *wnt8a* target loci would bind β-catenin yet remain refractive to transcriptional regulation by Wnt signalling in a different developmental context. For that reason we investigated whether the identified *wnt8a* target genes have any potential to respond to earlier maternal Wnt signalling (see Fig. S1).

**Fig. 4.**
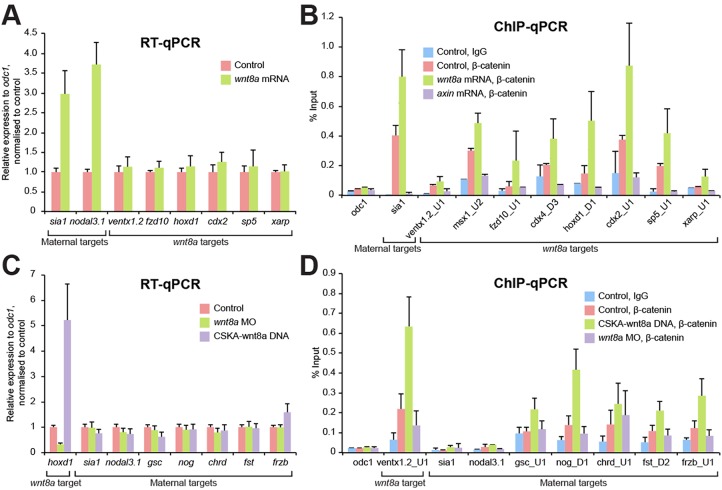
**β-catenin recruitment is not sufficient for transcriptional regulation.** (A,B) Maternally activated Wnt/β-catenin signalling regulates transcription of only context-specific maternal Wnt/β-catenin target genes. Experimental enhancement of maternal Wnt signalling, by injection of *wnt8a* mRNA at the two- to four-cell stage, increases expression of the maternal Wnt targets *sia1* and *nodal3.1* when analysed at the MBT, compared with the uninjected control (A). By contrast, expression levels of *wnt8a* target genes remain unchanged. However, β-catenin binding increases at both maternal Wnt target and zygotic *wnt8a*-regulated target loci at the 1000-cell stage (B). Note that the reduction of β-catenin binding following injection of *axin* mRNA indicates that maternally regulated endogenous β-catenin associates with not only maternal Wnt target genes but also zygotic *wnt8a* target genes. (C,D) Zygotically activated β-catenin controls the expression of only zygotic *wnt8a* targets. *wnt8a* MO or CSKA-wnt8a DNA were injected at the two- to four-cell stage and gene expression and β-catenin binding were analysed at the early gastrula stage. Knockdown of *wnt8a* reduces, and zygotic activation of Wnt8a signalling increases, expression of the *wnt8a* target *hoxd1*, as a control. Whereas *wnt8a* knockdown or overexpression does not affect the expression of maternal Wnt-regulated genes (C), overactivation of Wnt8a signalling increases β-catenin binding to some maternal Wnt-regulated loci (D) but not to the well-characterised direct maternal Wnt target genes *sia1* and *nodal3.1*. Error bars indicate s.d. and s.e.m. of three biological replicates for RT-qPCR and ChIP-qPCR, respectively.

We experimentally induced ectopic and enhanced activation of maternal Wnt signalling and examined the expression of several *wnt8a* target genes by RT-qPCR in blastula embryos at the MBT (Fig. 4A), as well as the known maternal Wnt targets *sia1* (Brannon et al., 1997) and *nodal3.1* (also known as *Xnr3*; McKendry et al., 1997) as controls. Enhanced activation of maternal Wnt signalling significantly increased expression of the maternal targets, as expected, but did not change expression of *wnt8a* target genes (Fig. 4A). This is consistent with the established idea that the *wnt8a* target genes represent ventral mesoderm-specific zygotic Wnt targets. However, β-catenin ChIP analysis revealed that, remarkably, β-catenin is associated with both maternal Wnt and *wnt8a* target gene loci in pre-MBT embryos (100-cell stage), when β-catenin is regulated by maternal Wnt signal and well before the onset of zygotic Wnt8a signalling (Fig. 4B, pink). Furthermore, the β-catenin occupancy increased with enhanced maternal Wnt activity (Fig. 4B, green). This observation was confirmed by pharmacological activation of maternal Wnt signalling activity with BIO (Fig. S7). β-catenin binding was reduced following experimental inhibition of endogenous maternal Wnt signalling (Fig. 4B, purple). This result clearly shows that maternal Wnt signalling controls β-catenin recruitment before the MBT not only to maternal Wnt target genes but also to *wnt8a* target loci. Thus, there appears no obvious qualitative difference in β-catenin recruitment between maternal Wnt target genes and *wnt8a* target genes.

Conversely, as expected, the transcription of genes known to be regulated by maternal Wnt signalling (Brannon et al., 1997; Crease et al., 1998; Wessely et al., 2001) remained unaffected by either *wnt8a* knockdown or experimentally enhanced Wnt signalling activity in gastrula embryos (Fig. 4C). However, β-catenin ChIP analysis in the same experiment revealed differences among maternal Wnt-regulated gene loci; for some (*gsc*, *nog*, *chrd*, *fst* and *frzb*), levels of β-catenin binding were increased by experimentally enhanced Wnt8a activity in gastrula embryos (Fig. 4D, green), similar to *wnt8a* targets (e.g. *ventx1.2*); whereas others (*sia1* and *nodal3.1*) were neither associated with endogenous β-catenin nor with experimentally activated β-catenin in gastrula embryos (Fig. 4D; see Table S4 for β-peaks of maternal Wnt-regulated gene loci). Together, these results demonstrate in two different developmental contexts that Wnt-regulated β-catenin association is not sufficient for transcriptional activation.

### Context-specific expression of *wnt8a* target genes is regulated by BMP and FGF signalling subsequent to β-catenin recruitment

**Fig. 5.**
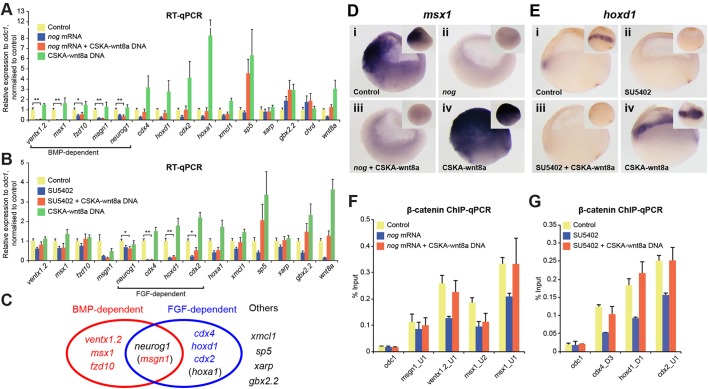
**BMP or FGF signalling is required for *wnt8a* target gene expression but not for β-catenin recruitment.** (A) BMP signalling is required for context-specific transcriptional regulation by Wnt8a signalling, but only of some *wnt8a* target genes. Two- to four-cell stage embryos were injected with BMP antagonist *noggin* (*nog*) mRNA. CSKA-wnt8a DNA was injected additionally to reinstate Wnt8a expression (as endogenous *wnt8a* expression is itself regulated by BMP signalling). Expression was analysed by RT-qPCR at the early gastrula stage. When BMP signalling is blocked, expression of BMP-dependent genes remains reduced even when Wnt8a expression is reinstated. (B) FGF signalling is required for context-specific transcriptional regulation by Wnt8a signalling, but only of some *wnt8a* target genes. Embryos were treated with the FGFR inhibitor SU5402 from the 1000/2000-cell stage through the early gastrula and injected where indicated with CSKA-wnt8a DNA at the two- to four-cell stages (to reinstate Wnt8a expression, as endogenous *wnt8a* expression is itself regulated by FGF signalling). When FGF signalling is inhibited, expression of FGF-dependent genes remains reduced, even when Wnt8a expression is reinstated. (C) *wnt8a* targets can therefore be classified into BMP-dependent or FGF-dependent genes. Note that some genes belong to both groups and others are neither BMP nor FGF dependent. (D,E) *In situ* hybridisation shows expression of *msx1* (D) and *hoxd1* (E) in sagittal sections and lateral views (insets) of control uninjected and experimentally manipulated embryos as indicated (dorsal to the right). (F) BMP signalling is not required for *wnt8a-*regulated β-catenin recruitment to BMP-dependent *wnt8a* target gene loci. Embryos were treated as in A and analysed by β-catenin ChIP-qPCR at the early gastrula stage. (G) FGF signalling is not essential for *wnt8a-*regulated β-catenin recruitment to FGF-dependent *wnt8a* target gene loci. Embryos were treated as in B and analysed by β-catenin ChIP-qPCR at the early gastrula stage. Uninjected, untreated embryos were used as controls in A,B,D-G. *P<0.1, **P<0.05; two-tailed Student's *t*-test. Error bars represent s.d. of four biological replicates (A,B) or s.e.m. of three biological replicates (F,G). Note that *wnt8a* gene expression itself was decreased by BMP or FGF pathway inhibition (*wnt8a* blue bars in A,B) but restored by co-injection of CSKA-wnt8a DNA (*wnt8a* orange bars in A,B) compared with controls (*wnt8a* yellow bars in A,B), and that higher *wnt8a* expression levels with CSKA-wnt8a DNA (*wnt8a* green bars in A,B) reflect both expression of endogenous *wnt8a* and expression from CSKA-wnt8a DNA, resulting in upregulation of several *wnt8a* target genes (in A,B).

Beyond the expected TCF/LEF motifs, *de novo* motif discovery among *wnt8a* target genes failed to identify further shared enriched motifs. We therefore sought to test earlier proposed hypotheses that combinatorial signalling underlies the context-specific expression of *wnt8a*-regulated genes. It has previously been suggested that, among our *wnt8a*-regulated genes, *ventx1.2* is co-regulated by BMP signalling (e.g. Hoppler and Moon, 1998). To investigate whether co-regulation by Wnt and BMP signalling represents a shared mechanism for regulating context-specific expression of *wnt8a* targets (reviewed by Itasaki and Hoppler, 2010), we examined the requirement of BMP signalling for *wnt8a* target gene regulation by blocking the BMP pathway while maintaining constant levels of Wnt8a signalling. We found that the expression of another four genes, in addition to *ventx1.2*, is dependent on BMP signalling (Fig. 5A), but, importantly, not that of all 13 genes in the shortlist. Thus, although decisive for context-specific expression of some genes in this tissue, BMP signalling is not an indispensable element of any general mechanism for context-specific regulation of *wnt8a* target genes.

Among our other *wnt8a* targets, *cdx4* had been shown to be co-regulated by FGF signalling (Haremaki et al., 2003). To examine whether other *wnt8a* targets are similarly co-regulated by FGF signalling, we analysed *wnt8a* target gene expression while inhibiting the FGF pathway under constant levels of Wnt8a signalling. Interestingly, compared with the BMP experiments, a largely distinct subset of *wnt8a* target genes was found to be FGF dependent (Fig. 5B). These results suggest that *wnt8a*-regulated genes can be categorised into at least two different groups based on co-regulation by different signalling pathways (Fig. 5C), and that there is therefore no collectively shared context-specific Wnt8a signalling mechanism that prevails in the ventral prospective mesoderm of gastrulae.

Since the BMP and FGF pathways are activated in different regions of early gastrulae (Fig. S8A) (Schohl and Fagotto, 2002), we examined whether contexts in which *wnt8a* target genes are regulated by these two pathways are spatially restricted. We performed whole-mount *in situ* hybridisation of several BMP-dependent or FGF-dependent *wnt8a* targets. Expression of the BMP-dependent *wnt8a* target gene *msx1* was detected in the prospective ectoderm and mesoderm and, as expected, it was significantly reduced in both tissues when BMP signalling was inhibited (Fig. 5D). Experimentally enhanced Wnt8a activity increased the expression in both tissues only when endogenous BMP signalling was active (similar results are shown for *fzd10* in Fig. S8B). On the other hand, the FGF-dependent *wnt8a* targets *hoxd1* (Fig. 5E) and *cdx2* (Fig. S8C) are expressed more exclusively in prospective mesoderm (the marginal zone). Blocking FGF signalling decreased their expression in prospective mesodermal cells. Activation of Wnt8a signalling did not reinstate their expression when FGF signalling was blocked, but did cause strongly induced expression of both genes when endogenous FGF signalling was active, specifically in the marginal zone. These results suggest that the BMP and FGF pathways provide different, spatially restricted contexts where *wnt8a* target genes can be activated in response to Wnt8a signalling; however, their respective spatially restricted contexts overlap in the prospective mesoderm.

We uncovered one shared aspect of gene regulation of these context-specific *wnt8a* targets. Since BMP and FGF signalling are required for normal *wnt8a* target gene regulation, we wondered whether these signalling mechanisms would regulate β-catenin recruitment to these *wnt8a* target loci. We observed, however, that β-catenin is still able to bind to *wnt8a* target loci at comparable levels to controls even when BMP or FGF signalling is inhibited, provided constant levels of Wnt8a signalling are maintained (Fig. 5F,G). This demonstrates that neither BMP nor FGF signalling restricts β-catenin recruitment to WREs. Rather, our results suggest that context-specific transcriptional regulation of *wnt8a* targets by the BMP or FGF pathway takes place in addition, and subsequent, to Wnt-regulated β-catenin binding to cis-regulatory sequences associated with these genes.

## DISCUSSION

### β-catenin is required but not sufficient for Wnt-regulated transcriptional activation

The interaction of nuclear β-catenin with target genomic loci has been shown to be sufficient to activate target gene transcription for many specific examples studied in a variety of tissues and experimental systems (recently reviewed by Zhang and Cadigan, 2014). Our results, however, demonstrate that chromatin association of β-catenin does not necessarily imply transcriptional activation. This is also consistent with data from a cell culture model of colorectal cancer demonstrating chromatin association of β-catenin near to many genes that are not regulated by β-catenin function (Watanabe et al., 2014). Our study is the first to investigate this phenomenon and to provide evidence that β-catenin binding to target loci can be Wnt regulated even in embryonic contexts, in which these genes are not transcriptionally Wnt responsive (Fig. 4). Furthermore, we uncover that molecular mechanisms (e.g. BMP or FGF pathways) required for context-specific transcriptional regulation of direct target genes do not influence the Wnt-regulated chromatin association of β-catenin (Fig. 5).

**Fig. 6.**
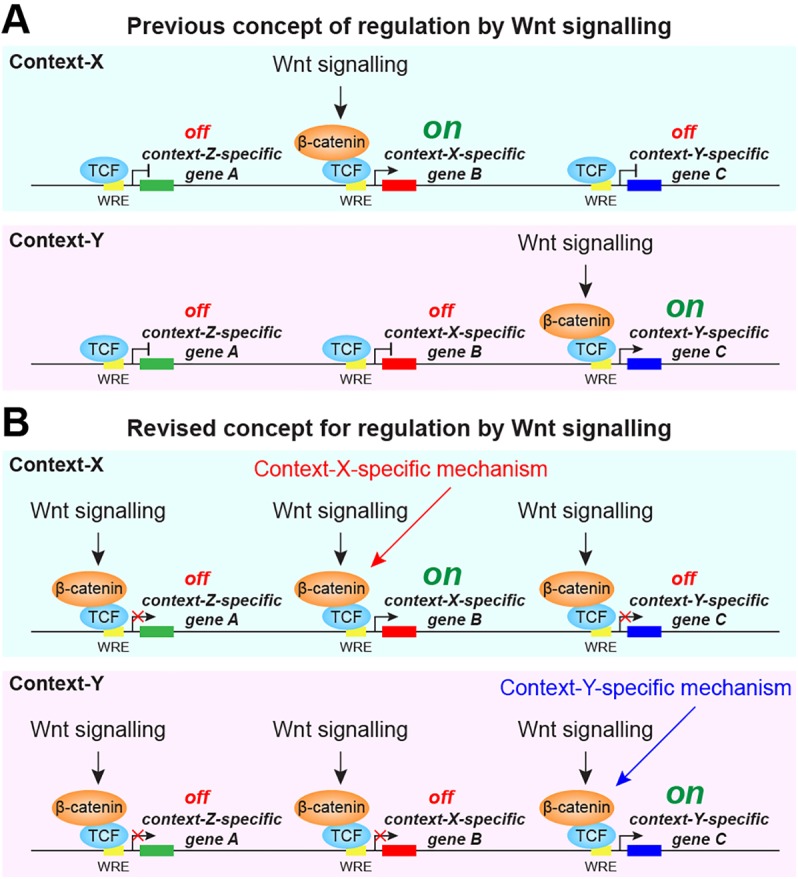
**Model for context-specific Wnt/β-catenin target gene regulation.** (A) In the previous concept established from studies of individual genes, Wnt signalling specifically controls β-catenin recruitment to the Wnt-response element (WRE) of context-specific target genes and leads to their transcription (e.g. *gene B* in the context-X). (B) In the revised concept from our studies, Wnt-regulated β-catenin recruitment takes place at numerous loci. Transcriptional activation at those loci is conditional on context-specific mechanisms (e.g. a context-X-specific mechanism for *gene B* in context-X).

These unexpected mechanistic findings suggest a more general paradigm for Wnt-regulated transcriptional mechanisms. Thus, chromatin association of β-catenin, even to functional WREs, is only productive for Wnt signalling-regulated transcriptional activation in the appropriate developmental context (Fig. 6). This new insight helps explain why we identify chromatin association of β-catenin near many genes that are not overtly transcriptionally regulated by the Wnt signalling mechanism operating at this stage (Fig. 3). Taken at face value, this would suggest that β-catenin ChIP-seq analysis is not of sufficient use on its own for detecting direct transcriptionally Wnt-regulated target genes, and it raises questions about the biological significance of apparently widespread β-catenin binding across the genome.

### Molecular mechanisms regulating context-specific Wnt/β-catenin target gene expression

Identifying direct Wnt8a/β-catenin targets was motivated by our ambition to uncover a unifying mechanism for context-specific Wnt/β-catenin target gene regulation in the ventrolateral prospective mesoderm. We wondered whether it would be possible to predict Wnt-regulated target genes from many β-catenin-bound loci. We found that *wnt8a* targets tend to show stronger and clearer β-catenin binding than non-*wnt8a*-regulated loci (Fig. 3E). While β-peaks generally appear to be enriched close to TSSs (as previously observed by Watanabe et al., 2014), this enrichment is even higher in confirmed *wnt8a* targets. These observations are consistent with the notion that transcriptionally regulated direct target genes exhibit high levels of transcription factor occupancy at nearby binding sites (Biggin, 2011). Although we anticipated that *wnt8a*-regulated genes would share specific DNA sequences under their β-catenin-bound regions, which we hoped would reveal a shared tissue-specific molecular regulatory mechanism, our sequence motif analysis suggested that only TCF/LEF-mediated mechanisms are shared (Fig. 3F). Individual β-catenin-associated genomic sequences contain consensus binding sequences for other transcription factors; however, such sequences are found both in the Wnt8a/β-catenin target genes that we identified and in non-*wnt8a*-regulated loci, and as such are not informative in the context of a shared tissue-specific molecular regulatory mechanism for Wnt/β-catenin target genes in the ventrolateral mesoderm. The presence of regulatory sequences for other transcription factors in some *wnt8a* target loci could indicate additional regulation of these genes, particularly by T-box transcription factors driving mesoderm induction and development in this tissue and at this stage (Gentsch et al., 2013). Overall, these trends do not add up to reliable criteria for predicting *wnt8a*-regulated genes from among all β-catenin-bound genomic loci, let alone Wnt transcriptionally regulated genes more generally.

Since direct target genes of maternal Wnt/β-catenin signalling were shown to be regulated by combinatorial Wnt and Smad2 (Activin/Nodal/TGFβ) signalling (Crease et al., 1998; Laurent et al., 1997), we hypothesized that context-specific *wnt8a* target genes shared an analogous common regulatory mechanism, possibly involving combinatorial signalling with another signalling mechanism. Indeed, we find that combinatorial signalling is important; however, more gene-specific mechanisms are unearthed: some *wnt8a* target genes are co-regulated by BMP, some by FGF signalling. The discovery of several classes of *wnt8a* target genes confirmed that there is no single, collectively shared tissue-specific mechanism for restricting Wnt/β-catenin target gene regulation in the context that we have investigated, and therefore studying these molecular mechanisms would not reveal shared ventral mesoderm-specific processes. This explains our inability to identify any shared motifs beyond potential TCF/LEF binding sequences. However, as expected, all *wnt8a* target genes co-regulated by BMP signalling contain potential Smad1 and Smad4 binding sequences, and all *wnt8a* targets co-regulated by FGF signalling contain potential ETS binding motifs.

### β-catenin binding to Wnt target genes in alternative contexts

Our β-catenin ChIP-seq analysis at the early gastrula stage found β-peaks at gene loci known to be transcriptionally regulated by maternal Wnt signalling at an earlier stage. However, when zygotic Wnt8a signalling is experimentally activated, β-catenin occupancy increases at these gene loci, but not gene expression. Conversely, we can detect β-catenin binding to *wnt8a* target loci even before the onset of endogenous *wnt8a* expression (Fig. 4). This precocious β-catenin binding to *wnt8a* target loci is regulated by maternal Wnt signalling, but this binding does not cause increased transcriptional expression. These results support our conclusion that chromatin association of β-catenin does not imply Wnt-regulated transcriptional activation and are therefore also consistent with context-specific regulatory mechanisms acting subsequent to Wnt-regulated β-catenin binding, as discussed above.

### Widespread distribution of β-catenin binding

Widespread binding to the genome is common for some DNA-binding transcription factors and is thought to be mediated via low-affinity sites (Biggin, 2011; MacQuarrie et al., 2011). However, β-catenin association does not, on the whole, result from indiscriminate binding across the genome but rather β-catenin tends to be recruited to putative CRMs (promoter and enhancer sequences). In particular, we find a significant level of overlap between our β-peaks and TLE ChIP-seq peaks, which have recently been found to be indicators of tissue-specific CRMs (Yasuoka et al., 2014).

β-catenin is reported to be predominantly associated with TCF/LEF motif-containing chromatin, both in cancer cells with activated Wnt signalling (Schuijers et al., 2014; Watanabe et al., 2014) and Wnt-induced embryonic stem cells (Zhang et al., 2013). Our analysis also identified TCF/LEF as the only shared sequence motif among the validated 13 direct *wnt8a* target genes, suggesting that positive gene regulation by Wnt/β-catenin signalling is mediated by TCF/LEF-dependent mechanisms. However, our *de novo* motif search revealed that non-*wnt8a*-regulated, yet β-catenin-bound, loci also contain consensus binding sequences for transcription factors other than TCF/LEF, suggesting that some β-catenin protein may interact with those transcription factors when associated with genomic sequences of non-*wnt8a*-regulated genes. In fact, such interactions have previously been reported for OCT4 (POU5F1) (Kelly et al., 2011), TBX5 (Rosenbluh et al., 2012), SOX proteins (Kormish et al., 2010) and FOX proteins (Zhang et al., 2011); and, among them, OCT4 (Abu-Remaileh et al., 2010) and SOX proteins (Kormish et al., 2010) are known to negatively regulate β-catenin-dependent transcriptional regulation. Thus, the β-catenin-bound yet non-*wnt8a*-regulated gene loci identified in our analysis might be deliberately repressed by these transcription factors. Alternatively, chromatin association of β-catenin via these transcription factors might act as part of a buffering system to fine-tune the availability of β-catenin for transcriptional regulation at Wnt/β-catenin target genes, similar to that previously suggested for fine-tuning the availability of functional DNA-binding transcription factors (MacQuarrie et al., 2011). In particular, our analysis of motifs enriched in β-peaks close to non-*wnt8a*-regulated loci identifies the same combined SOX and OCT4 motif that has previously been reported in embryonic stem cell studies (Zhang et al., 2013). Although technical bias cannot currently be excluded, the β-catenin chromatin association observed in our *Xenopus* embryos seem more similar to that of embryonic stem cells than cancer cells (Schuijers et al., 2014; Watanabe et al., 2014). Future analysis might confirm that β-catenin association with chromatin containing the combined SOX and OCT4 motif in particular is specifically prevalent in embryonic cells.

### Wnt/β-catenin target genes in the genome

Our results do not allow us to rule out the possibility that low levels of nuclear β-catenin associate with chromatin to mediate other, as yet undiscovered functions for β-catenin in the genome or to be part of a buffering system to fine-tune the availability of β-catenin for transcriptional regulation, as mentioned above. β-catenin-bound, yet non-*wnt8a*-regulated, gene loci in our analysis could more generally represent real Wnt target genes, but those that are regulated by Wnt signalling in other tissues and at other stages. Consistent with this idea, the GO analysis suggests that such genes are more associated with functions at later stages of development, after the stage of our analysis in early gastrulation, such as neural development, and also with metabolism (Fig. 3D). As a particular example, *sall4*, which is among our β-catenin-bound but not our *wnt8a*-regulated genes, has recently been identified as a direct *wnt3a* target gene during neural development (Young et al., 2014). In addition, our *wnt8a* target *msx1* showed a β-peak (msx1_U2) that is located at a conserved limb bud-specific enhancer (Miller et al., 2007), consistent with our hypothesis that β-catenin recruitment already occurs during early embryonic stages to cis-regulatory elements responsible for Wnt-mediated regulation in other tissues at later stages. Furthermore, ∼60% of orthologues of the Wnt target genes listed at the curated Wnt homepage (http://web.stanford.edu/group/nusselab/cgi-bin/wnt/) are represented in our list of β-catenin-bound genes. Likewise, our list contains 70% of homologues of direct β-catenin-regulated target genes identified in a colorectal cancer cell line (Watanabe et al., 2014). Therefore, many potential direct Wnt targets in the genome could be associated with β-catenin, even if their expression is not Wnt regulated in the tissue analysed.

### Conclusions

Our investigation challenges the fundamental concept that β-catenin recruitment to individual Wnt target genes predictably drives transcriptional expression (Fig. 6); instead, it introduces a more general paradigm for Wnt-regulated transcriptional mechanisms, which is more relevant for the repeated and tissue-specific functions of Wnt/β-catenin signalling in embryonic development, stem cell-mediated homeostasis and cancer. We discovered that chromatin association of β-catenin, even to functional WREs, does not imply transcriptional activation. Wnt signalling regulates β-catenin binding to target loci even in embryonic contexts, in which these gene loci are not transcriptionally Wnt responsive. Chromatin association of β-catenin is only productive for Wnt signalling-regulated transcriptional activation in the appropriate developmental context. Mechanisms regulating this developmental context therefore do not necessarily influence the Wnt-regulated association of β-catenin with chromatin. Our findings will also be relevant beyond early embryogenesis, with implications for cancer research and other Wnt-related diseases, where an abnormal subtle change in cellular context may induce the anomalous expression of genes, with deleterious consequences.

## MATERIALS AND METHODS

### Embryo experiments

*Xenopus tropicalis* (Gray, 1864) embryos were obtained by natural mating of adult males and females or by *in vitro* fertilisation as described by del Viso and Khokha (2012) and staged according to Nieuwkoop and Faber (1967). The fertilised embryos were injected with MOs and mRNAs, and treated with chemical inhibitors as indicated, and then cultured in 0.1× Marc's Modified Ringer (MMR) at 28°C. MOs (Gene Tools) were: CoMO, 5′-CCTCTTACCTCAGTTACAATTTATA-3′ (Heasman et al., 2000); *wnt8a* MO, 5′-GGAGACTGCTATCCAGGGTAATGCT-3′ (Rana et al., 2006). pCSKA-wnt8a was created as a *wnt8a* MO-insensitive *wnt8a* gene by introducing nucleotide substitutions (Fig. S3A). Capped mRNA was synthesized using the mMESSAGE mMACHINE Kit (Ambion) and the following linearised DNA templates were used: pCS2+ Xwnt-8, Axin/CS2mt and pCS2+ noggin. SU5402 (SML0443) was purchased from Sigma. See the supplementary Materials and Methods for further details of *Xenopus* embryos and treatment with MOs, mRNAs and chemical inhibitors.

### Whole-mount RNA *in situ* hybridisation

Digoxigenin-labelled antisense RNA probes were synthesized from linearised template plasmids (see the supplementary Materials and Methods) using the MEGAscript Transcription Kit (Life Technologies) for use in whole-mount RNA *in situ* hybridisation as described by Lavery and Hoppler (2008).

### RT-PCR

Total RNA was isolated from whole embryos as described by Lee-Liu et al. (2012). cDNA was synthesized using the QuantiTech Reverse Transcription Kit (Qiagen).

### qPCR

qPCR was performed using a LightCycler 480 and SYBR Green I Master reagents (Roche). For RT-qPCR, relative expression levels of each gene to *odc1* were calculated and then normalised to the control. For primer sequences for RT-qPCR and ChIP-qPCR, see the supplementary Materials and Methods.

### ChIP

ChIP analysis was carried out as described (Akkers et al., 2012; Blythe et al., 2010; Janssens et al., 2010) with slight modifications: after homogenisation, embryos were sonicated with a Bioruptor Plus (Diagenode). Two β-catenin antibodies, namely anti-*Xenopus* β-catenin antibody (1:28; Blythe et al., 2009) and anti-β-catenin antibody [1:28 (2 µg); H-102; sc-7199, Santa Cruz Biotechnology], and normal rabbit IgG [1:56 (2 µg); sc-2027, Santa Cruz Biotechnology] were used for immunoprecipitation. For optimised conditions of the β-catenin ChIP experiment, see Fig. S2 and the supplementary Materials and Methods.

### ChIP-seq

β-catenin ChIP was performed using anti-β-catenin antibody (H-102) as described above. Two Illumina TrueSeq ChIP libraries were constructed from ChIP and input control DNA and sequenced using Illumina HiSeq 2500 at The Genome Analysis Centre (TGAC, Norwich, UK). Sequenced reads were mapped to the *X*. *tropicalis* genome assembly JGI 4.2. Briefly, MACS2 (Zhang et al., 2008) and SPP (Kharchenko et al., 2008) were used for peak calling. Reproducible peaks were identified using the IDR method (Li et al., 2011). Peaks were assigned to closest genes using the distanceToNearest function [rtracklayer (Lawrence et al., 2009) and GenomicRanges (Lawrence et al., 2013)]. Heat maps were created using HOMER, Cluster 3.0, and Java Treeview. Histograms were visualised using HOMER and Excel. *De novo* motif discovery was performed using MEME-ChIP. ChIP-seq and RNA-seq data were visualised on the UCSC genome browser. GO analysis was performed using the PANTHER classification system (Mi et al., 2013). We carried out statistical over-representation tests using PANTHER GO annotations (PANTHER GO-Slim Biological Process, PANTHER Protein Class, PANTHER Pathways). See the supplementary Materials and Methods for details. The ChIP-seq data sets are available in GEO under the accession number GSE72657.

### RNA-seq

Total RNA was extracted as described by Lee-Liu et al. (2012). Illumina TruSeq RNA libraries were constructed and sequenced using Illumina HiSeq 2000 at TGAC. Sequenced reads were aligned to the *X*. *tropicalis* genome JGI 4.2 with gsnap. Aligned reads were counted using HTSeq (Anders et al., 2015) and further differential gene expression analysis was carried out using DESeq2. See the supplementary Materials and Methods for details. The RNA-seq data sets are available in GEO under the accession number GSE72657.

### Reporter constructs and luciferase assay

Genomic fragments of β-peaks were amplified by PCR and subcloned into the pGL4.10 vector (Promega) or a derivative vector pβ-actin-luc carrying a heterologous basal promoter. Embryos were injected with 40 pg reporter plasmid DNA together with 40 pg pRL-CMV (Promega) at the two- to four-cell stage, collected at the early gastrula stage, and assayed for luciferase activity. For cloning into luciferase reporter constructs, see the supplementary Materials and Methods.
